# On the Means of Administering Food to Insane Persons, in Cases of Resistance

**Published:** 1827-10

**Authors:** Caleb Williams

**Affiliations:** Surgeon to the Retreat, York.


### INSANITY.

*On the Means of administering Food to Insane Persons, in Cases of Resistance.* By Caleb Williams, Surgeon to the Retreat, York.

Those who have had but little intercourse with the insane, must have witnessed some of those unhappy cases where the patient refuses to take his food, and where every persuasion and artifice prove alike unavailing in breaking through the morbid chain of ideas, or removing his erroneous scruples. In such cases it is of great importance that the food should be conveyed into the stomach with as little injury or suffering to the unfortunate patient as possible: for this purpose numerous expedients have been tried, many of which have very imperfectly accomplished the object; whilst others, under certain circumstances, are not free from danger even to the life of the individual, and may fail in the chief object, that of carrying food into the stomach.

Soon after I was appointed surgeon to the Retreat, (an establishment for insane persons of the Society of Friends near York,) I was present at the feeding of a female patient. On this occasion the boat was used, which is an oval tin vessel, having a broad flattened tube or spout, an inch wide and a quarter of an inch deep: the patient being properly secured in the rocking-chair, the extremity of this tube, wrapped with linen, was introduced into the mouth, the nose being at the same time pressed, and the food conveyed to the entrance of the throat. In this case the determination not to swallow was so fixed and resolute, that all means were unavailing, notwithstanding the suffering which she must have endured from holding her breath, to induce her to swallow the food which was thus forced to the back part of the mouth. The face having become extremely livid, the vessels of the eyes quite turgid with blood, I requested the attendants to desist when she seemed almost exhausted from the violent effort she had been obliged to make to prevent the passage of the food.

Under these circumstances it was important to try some other plan, which should with certainty convey nourishment into the stomach, and at the same time incur no risk of personal injury. It occurred to me that an œsophagus tube possessed these advantages, and that an elastic gum bottle, with a stop-cock attached to it, would form a pretty complete apparatus for the purpose. The mouth was opened by means of a small wooden lever, seven or eight inches long, and tapering off to a blunt point, which was introduced between the teeth, and, being carried to the side of the mouth, enabled me to open it as far as necessary. The œsophagus tube was then easily pressed into the pharynx, and down into the stomach; a small piece of wood, perforated in the middle to admit the extremity of the tube, was then placed in the mouth, to keep it open, and prevent the teeth from compressing or dividing the tube; the elastic-gum bottle, previously filled, and closed by the stop-cock, was adjusted to the end of the tube, and its contents, without any difficulty, conveyed into the stomach. This operation appeared to occasion very little inconvenience, and was repeated two or three times a-day: since that time Read's syringe has been used instead of the elastic-gum bottle, and has been found well calculated for the purpose. In this way another female patient was fed two or three times a-day for upwards of four months, without any personal injury or material suffering.

It is proper to remark, however, that the œsophagus-tubes in Read's cases are much larger than is necessary for the purpose, and consequently create much greater uneasiness to the patient; the smaller ones, made of elastic gum, I think are preferable. The "guard," or piece of wood with a hole in the centre, contained also in Read's cases, serve very well to keep the patient's mouth open, and prevent pressure on the tube.

